# Investigation of serum cortisol concentration as a potential prognostic marker in hospitalized dogs: a prospective observational study in a primary care animal hospital

**DOI:** 10.1186/s12917-019-1919-4

**Published:** 2019-05-24

**Authors:** Masashi Yuki, Reina Aoyama, Takashi Hirano, Reina Tawada, Mizuho Ogawa, Eiji Naitoh, Daiki Kainuma, Noriyuki Nagata

**Affiliations:** Yuki Animal Hospital, 2-99 Kiba-cho, Minato-ku, Aichi Japan

**Keywords:** Cortisol, Dogs, Medical disease, Prognostic

## Abstract

**Background:**

Dogs with various medical diseases are usually treated at hospitals; however, the prognostic markers in dogs remain unknown. The aim of this study was to investigate the ability of serum cortisol concentration (SCC) to predict the prognosis of dogs with medical diseases. At 0 and 24 h after hospitalization, the neutrophil count, lymphocyte count, blood glucose concentration, and SCC were measured. Survival for 30 days from the time of hospitalization was investigated, and the dogs were divided into a survivor group and a non-survivor group.

**Results:**

The neutrophil count at 24 h, SCC at 24 h, increase in SCC from 0 to 24 h (Inc-SCC), and the rate of increase in SCC from 0 to 24 h (R-Inc-SCC) were significantly higher in the non-survivor group than in the survivor group. The area under the receiver operating characteristic (ROC) curve values for the neutrophil count at 24 h, SCC at 24 h, Inc-SCC, and R-Inc-SCC were 0.695, 0.72, 0.63, and 0.66, respectively. Using the highest area under the ROC curve value, the sensitivity and specificity of SCC at a cutoff level of 6.6 μg/dL for predicting mortality were 89.5 and 61.9%, respectively. Moreover, the Kaplan–Meier curves confirmed the significant prognostic influence of SCC at 24 h.

**Conclusions:**

SCC as a marker of stress is a useful biomarker for predicting the prognosis of dogs with medical diseases requiring hospital treatment.

## Background

Primary care animal hospitals treat various medical diseases in dogs. However, to date, there have been no known prognostic biomarkers that can be applied to various medical diseases in hospitalized dogs.

In a number of physiologically stressful conditions, including critical illnesses, the hypothalamic–pituitary–adrenal (HPA) axis is generally activated through extensive immune-neuroendocrine interactions; this activation of the HPA axis has been well demonstrated among previous studies [1–11]. The methods of evaluating stress levels in human patients and dogs include measuring the HPA axis hormones, most commonly cortisol concentrations [7, 8, 12–18]. The measurement of cortisol in the saliva was also attempted in human patients and dogs [19–22]; however, sample collection is more difficult in small dogs than in humans.

In recent studies on humans, it was found that the total and free serum cortisol concentrations increased with increasing severity of illness and were positively correlated with mortality [4, 12, 13, 16, 18, 23]. The concentration of cortisol has also been reported to be a useful prognostic marker [16, 18, 23–25]. Therefore, we examined the ability of serum cortisol concentration (SCC) as a stress marker to predict the prognosis of dogs with various medical diseases requiring admission at a primary care animal hospital. A notable feature of this study was that the data represented early-stage disease and were not from a referral hospital population with varying durations of illness.

## Methods

### Patient population

In this prospective observational study, 238 dogs with various diseases requiring hospitalization at Yuki Animal Hospital between February 2016 and May 2017 were recruited. Initially, cases that required surgery, those discharged within 24 h and insufficient data were excluded. Subsequently, cases diagnosed with hyperadrenocorticism and those treated with glucocorticoids or ketoconazole, which can affect the SCC, were excluded [16]. The remaining 67 dogs with medical diseases were included in this study.

The criteria for hospitalization were defined by physical examinations, complete blood count (CBC), serum biochemistry profiles, radiography, ultrasonography, or other examinations. All hospitalized cases were treated at the hospital for > 24 h. Samples collected at 0 and 24 h after hospitalization were analyzed for neutrophil count, lymphocyte count, blood glucose concentration, SCC, and C-reactive protein (CRP) concentration. The samples obtained in this study were used with the consent of the owner.

### Blood collection and quantification

Blood samples for CBC, serum, and plasma measurements were collected from all dogs via venipuncture of the cephalic, saphenous, or jugular vein and were placed in tubes with or without an anticoagulant. These samples were collected twice. The first sample was collected between 8:30 AM and 8:30 PM, and the second sample was collected 24 h later. Tubes with serum or plasma were centrifuged within 30 min of collection to separate the components.

### Assays

**Fig. 1:**
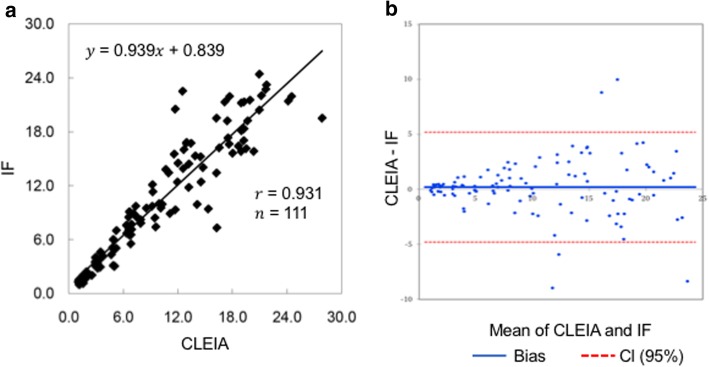
The measured values showed a very good correlation with the values determined by the CLEIA (**a**), and this agreement was confirmed by Bland–Altman analysis (**b**). CLEIA, chemiluminescent enzyme immunoassay; IF, immunofluorescence

CBC was measured using an automated hematology analyzer.^1^ The serum biochemical profile, including the CRP concentration, was obtained using a dry chemistry analyzer.^2^ Likewise, the SCC was obtained using a dry chemistry analyzer^3^ using immunofluorescence methods. The intra- and interassay coefficient of variation values were 2.58% (mean: 9.36 μg/dL) and 2.77% (mean: 8.88 μg/dL), respectively. The measured values showed a very good correlation with the values determined by the chemiluminescent enzyme immunoassay method^4^ (*y* = 0.939 *x* + 0.839, *r* = 0.931, *n* = 111 for SCC) (Fig. 1a), and this agreement was confirmed by Bland–Altman analysis (Fig. 1b). The range of the correlation data was 1.05–27.9 μg/dL, and the measurement range was 1.0–30.0 μg/dL. In healthy dogs with available CBC and biochemical profile (*n* = 37), the median SCC with a reference interval was 3.9 μg/dL (interquartile range [IQR]: 2.6–5.6 μg/dL).

### Study endpoints

In this study, the follow-up period was 30 days from the day of hospitalization. The outcome (survival or non-survival) was investigated on the basis of interviews with the dog owners and a review of the medical records.

### Analysis of stress markers

At 30 days after hospitalization, the dogs were divided into two groups as follows: a survivor group (*n* = 42) and a non-survivor group (*n* = 25). The neutrophil count, lymphocyte count, blood glucose concentration, CRP concentration, SCC at 0 and 24 h, age, sex, body weight, increase in SCC from 0 to 24 h (Inc-SCC), and rate of increase in SCC from 0 to 24 h (R-Inc-SCC) were investigated and compared between the two groups.

A receiver operating characteristic (ROC) curve analysis was performed for the parameters with significant differences between the survivor and non-survivor groups. Thereafter, the area under the curve, cutoff values, sensitivity, and specificity were evaluated. Finally, univariate and multivariate analyses of the factors affecting survival were performed.

### Statistical analysis

The neutrophil count, lymphocyte count, blood glucose concentration, SCC at 0 and 24 h, age, body weight, Inc-SCC, and R-Inc-SCC were compared between the two groups using the Mann–Whitney *U* test. Sex was compared between the two groups using Fisher’s exact test. The accuracy of the tests was assessed using ROC analysis. The area under the ROC curve, as well as the sensitivity and specificity with 95% confidence intervals (CIs), was calculated based on the outcome (survivor and non-survivor) of 30 days. Survival curves were generated using the Kaplan–Meier product-limit method and were compared using the log-rank test. Univariate (Fisher’s exact test) and multivariate (logistic regression analysis) analyses were performed on factors that were likely to affect mortality. The effect size was calculated as a power analysis. Statistical analyses were performed using the software Easy R [26]. Values with *p* < 0.05 were considered statistically significant.

## Results

### Patient population

In total, 67 dogs were enrolled in this study. These dogs included 28 females (7 sexually intact, 21 spayed) and 39 males (19 sexually intact, 20 neutered). Their median age was 12 years (*n* = 66, IQR: 10–14 years), and their median body weight was 4.0 kg (*n* = 67, IQR: 3.0–7.3 kg). Date about the dog breeds have been summarized in Table 1.

**Table 1:** Breeds and number of dogs

Breeds	No.
Miniature Dachshund	17
Chihuahua	12
Toy Poodle	8
Yorkshire Terrier	6
Mixed breed	6
Shih Tzu	3
American Cocker Spaniel	2
Papillon	2
Pomeranian	2
Basenji	1
Basset Hound	1
Golden Retriever	1
Great Pyrenees	1
Maltese	1
Miniature Bull Terrier	1
Miniature Pinscher	1
Pekinese	1
Shiba Inu	1

Table 2 shows the baseline characteristics of the study population. The most frequently diagnosed diseases were pancreatitis (*n* = 12), mitral valve disease (*n* = 8), and cholangiohepatitis (*n* = 7). The disposition of the survivor and non-survivor groups is likewise shown in Table 3. The median duration of hospitalization was five days (IQR: 5–8 days). In the non-survivor group, the median survival time was three days (*n* = 25, IQR: 3–6 days); one of the dogs was euthanized on the eighth day.

**Table 2:** Categorization according to medical condition and disposition of survivor and non-survivor group

Diagnosis	Total (no.)	Survivors (no.)	Non-survivors (no.)
Gastrointestinal disease			
Pancreatitis	12	10	2
Cholangiohepatitis	7	7	0
Gastroenteritis	4	3	1
Hepatic abscess	1	1	0
Urologic disease			
Acute/Chronic kidney disease	5	1	4
Bacterial cystitis	3	2	1
Ureter/Bladder calculus	2	2	0
Prostatitis	2	2	0
Pyelonephritis	1	1	0
Circulatory disease			
Mitral valve disease	8	5	3
Endocrine disease			
Diabetes mellitus	5	2	3
Hypercalcemia	3	2	1
Respiratory disease			
Pneumonia	2	0	2
Mediastinal emphysema	1	1	0
Nervous disease			
Vestibular disease	1	0	1
Immune-mediated disease			
Immune-mediated hemolytic anemia	1	0	1
Skin disease			
External otitis	1	1	0
Unknown diagnosis	8	2	6

**Table 3:** Comparison of age, sex, body weight, and the results of laboratory tests between survivor and non-survivor groups

Variable	RI	Survivor group (no., IQR)	Non-suvivor group (no., IQR))	*p* value
Age, median (years)		12.1 (n = 41, 10.7-13.5)	12.8 (n = 25, 10.1-15.3)	0.38
Sex				
Male		n = 22	n = 17	0.30
Female		n = 20	n = 8	
B.W. (kg)		3.8 (n = 42, 2.7-6.5)	5.3 (n = 25, 3.2-8.1)	0.15
Neu (/μL)	4,300–9,100			
0 hour		11,800 (n = 41, 8,200-17,200)	16,400 (n = 25, 10,600-19,300)	0.12
24 hour		12,800 (n = 38, 9,225-16,800)	17,950 (n = 20, 13,750-22,550)	0.01
Lym (/μL)	2,000–4,600			
0 hour		2,000 (n = 41, 1,500-3,100)	3,000 (n = 25, 1,700-4,800)	0.11
24 hour		1,950 (n = 38, 1,300-3,150)	2,200 (n = 20, 1,650-4,525)	0.14
Glu (mg/dL)	75.0–128.0			
0 hour		100.5 (n = 42, 91.8-118.0)	109.0 (n = 24, 86.3-136.0)	0.84
24 hour		108.0 (n = 35, 97.5-116.5)	120.5 (n = 20, 100.3-160.3)	0.53
CRP (mg/dL)	0–1.0			
0 hour		7.4 (n = 37, 2.7-17.1)	9.8 (n = 23, 2.6-17)	0.84
24 hour		8.4 (n = 25, 4.5-14.1)	16.5 (n = 12, 8.4-19.1)	0.31
SCC (μg/dL)	1.0–6.0			
0 hour		7.5 (n = 42, 4.6-18.3)	12.1 (n = 25, 6.3-22.9)	0.20
24 hour		4.8 (n = 42, 3.7-8.6)	8.8 (n = 20, 7.1-15.8)	< 0.001
Inc SCC (μg/dL)		-2.9 (n = 42, -10.6-0)	-0.25 (n=20, -5.8-1.5)	0.02
R Inc SCC (%)		0.51 (n = 42, 0.34-1)	0.99 (n = 20, 0.57-1.2)	< 0.001

*RI* reference intervales, *IQR* interquartile range, *B.W.* body weight, *Neu* neutrophile count, *Lym* lymphocyte count, *Glu* blood glucose concentration, *CRP* C reactive protein, *SCC* serum cortisol concentration, *Inc SCC* increase in SCC from 0 h to 24 h, *R Inc SCC* rate of increase in SCC from 0 h to 24 h

*p* values < 0.05 were considered statistically significant

### Analysis of stress markers

At 0 h, the median values were 12,900 cells/μL (*n* = 66, IQR: 8850–18,100 cells/μL) for neutrophil count; 2500 cells/μL (*n* = 66, IQR: 1500–3700 cells/μL) for lymphocyte count; 104.0 mg/dL (*n* = 66, IQR: 90.3–131.5 mg/dL) for blood glucose concentration; 8.9 mg/dL (*n* = 60, IQR: 2.7–17.3 mg/dL) for CRP concentration; and 10.8 μg/dL (*n* = 67, IQR: 5.0–21.2 μg/dL) for SCC. At 24 h, the median values of neutrophil count, lymphocyte count, blood glucose concentration, CRP concentration, and SCC were 14,250 cells/μL (*n* = 58, IQR: 10,000–19,375 cells/μL), 1950 cells/μL (*n* = 58, IQR: 1300–3400 cells/μL), 111.0 mg/dL (*n* = 55, IQR: 97.5–127.0 mg/dL), 10.0 mg/dL (*n* = 37, IQR: 4.5–17.5 mg/dL), and 7.1 μg/dL (*n* = 62, IQR: 3.9–9.4 μg/dL), respectively.

Comparisons of the median neutrophil count, lymphocyte count, blood glucose concentration, CRP concentration, SCC, Inc-SCC, and R-Inc-SCC between the survivor and non-survivor groups are shown in Table 3. Compared with the survivor group, the non-survivor group had a significantly higher neutrophil count at 24 h (*p* = 0.01), SCC at 24 h (*p* < 0.001), Inc-SCC (*p* = 0.02), and R-Inc-SCC (*p* < 0.001).

**Fig. 2:**
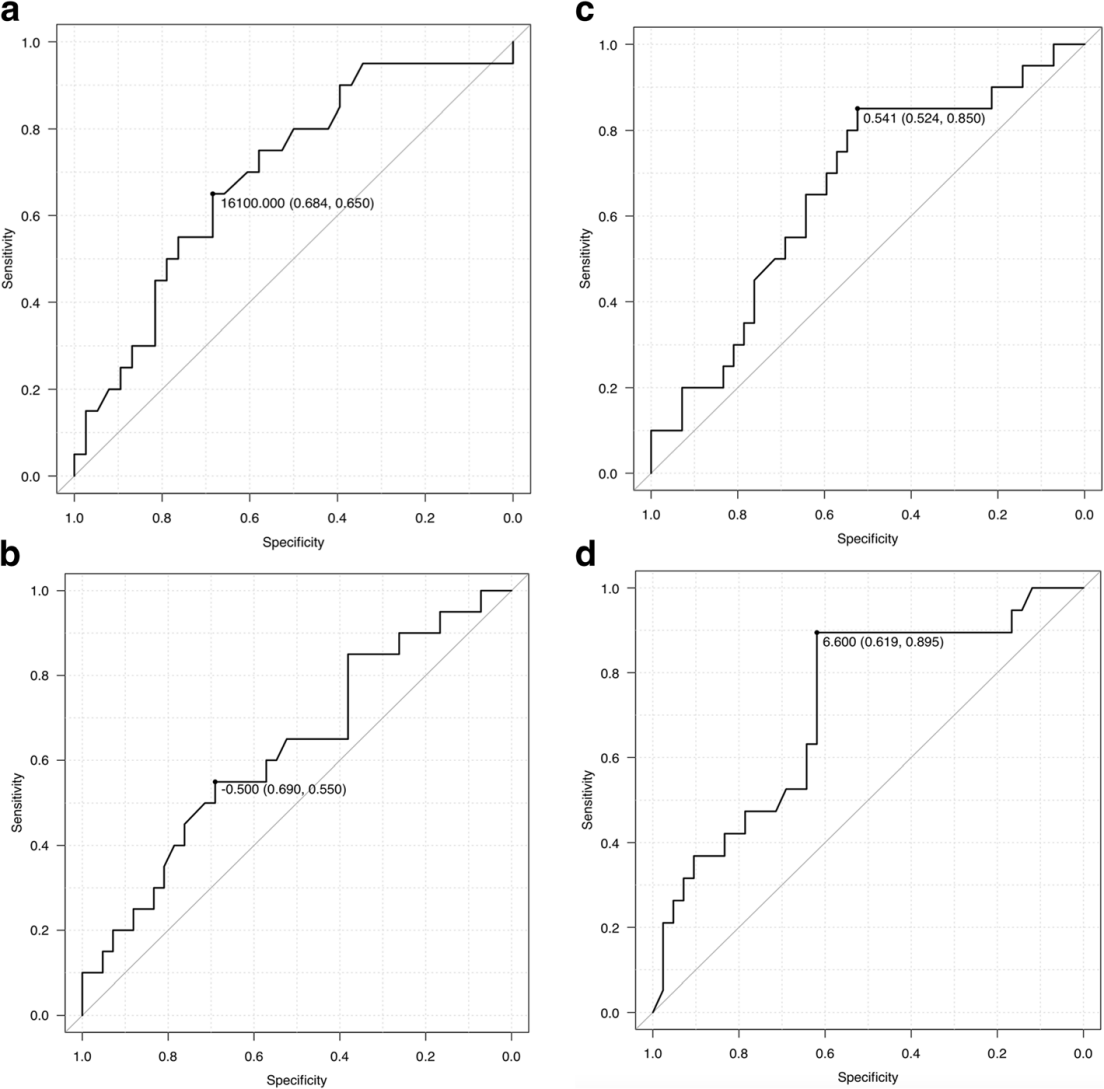
ROC curve for the neutrophil count at 24 h, Inc-SCC, R-Inc-SCC, and SCC at 24 h, plotted against survival after 30 days. The area under the ROC curve values of the neutrophil count at 24 h, Inc-SCC, R-Inc-SCC, and SCC at 24 h are 0.695 (95% CI: 0.55–0.83) (**a**), 0.63 (95% CI: 0.47–0.78) (**b**), 0.66 (95% CI: 0.51–0.81) (**c**), and 0.72 (95% CI: 0.58–0.86) (**d**), respectively. The sensitivity and specificity of SCC at 24 h (cutoff: 6.6 μg/dL) in predicting mortality are 89.5 and 61.9%, respectively (**d**). Inc-SCC, increase in serum cortisol concentration; ROC, receiver operator characteristic; R-Inc-SCC, rate of increase in serum cortisol concentration; SCC, serum cortisol concentration

The area under the ROC curve values showed the highest value in SCC at 24 h, and the diagnosability was the best (Fig. 2 a-d). The area under the ROC curve of SCC at 24 h was 0.72 (95% CI: 0.58–0.86) (Fig. 2d). The sensitivity and specificity of SCC at 24 h in predicting mortality (cutoff: 6.6 μg/dL) using the highest area under the ROC curve value were 89.5 and 61.9%, respectively (Fig. 2d).

**Fig. 3:**
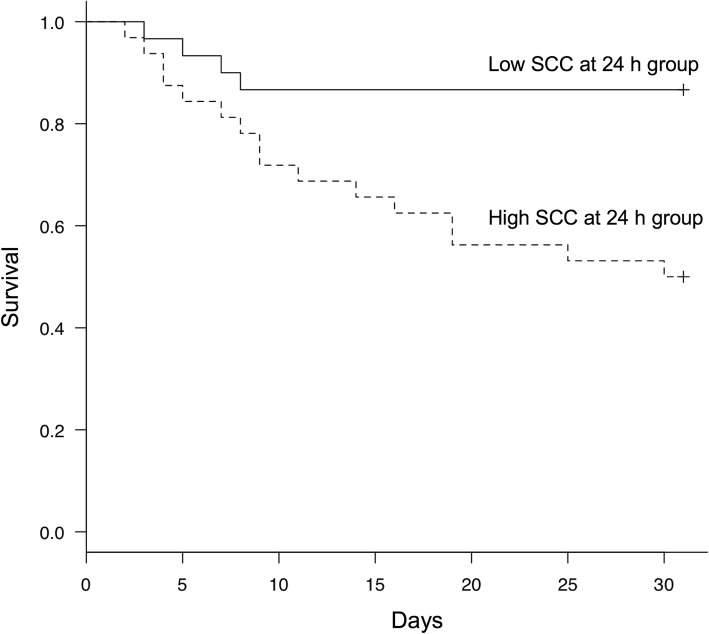
Kaplan–Meier curves of SCC at 24 h. The Kaplan–Meier curves and log-rank test confirm the significant influence of SCC at 24 h on the prognosis of dogs hospitalized for medical diseases (*p* < 0.001). SCC, serum cortisol concentration

The Kaplan–Meier curves and log-rank tests confirmed the significant prognostic influence of SCC at 24 h (*p* < 0.001) (Fig. 3). The univariate analysis identified higher values of R-Inc-SCC (*p* = 0.03) and SCC at 24 h (*p* = 0.01) as significant factors for mortality (Table 4). The multivariate analysis identified higher SCC at 24 h (odds ratio [OR]: 4.93; 95% CI: 1.26–19.2; *p* = 0.02] as a significant predictor of mortality. However, age (OR: 1.14; 95% CI: 0.31–4.18; *p* = 0.84) and neutrophil count at 24 h (OR: 2.71; 95% CI: 0.77–9.57; *p* = 0.12) were not significant predictors of mortality (Table 4). Based on the results obtained through this study, the effect size was *h* = 0.75.

**Table 4:** Univariate and multivariate analysis

	Univariate analysis		Multivariate analysis	
Variable	*p* value	OR	95% CI	*p* value
Age	0.31	1.14	0.31-4.18	0.84
Neu (/μL)				
0 hour	0.31			
24 hour	0.05	2.71	0.77-9.57	0.12
Lym (/μL)				
0 hour	0.13			
24 hour	1.0			
Glu (mg/dL)				
0 hour	0.20			
24 hour	0.27			
CRP (mg/dL)				
0 hour	0.43			
24 hour	0.72			
SCC (μg/dL)				
0 hour	0.21			
24 hour	0.013	4.93	1.26-19.2	0.02
Inc SCC (μg/dL)	0.42			
R Inc SCC (%)	0.03			

*OR* odds ration, *CI* confidence interval, *Neu* neutrophile count, *Lym* lymphocyte count, *Glu* blood glucose concentration, *CRP* C reactive protein, *SCC* serum cortisol concentration, *Inc SCC* increase in SCC from 0 h to 24 h, *R Inc SCC* rate of increase in SCC from 0 h to 24 h

*p* values < 0.05 were considered statistically significant

## Discussion

The results of this study suggested that SCC at 24 h after hospitalization is a useful prognostic marker of various medical diseases in dogs that require hospitalization. Although the SCC had an area under the ROC curve value of 0.72, which indicated a moderate accuracy, it was considered to be a sufficient marker to predict mortality. The survival rate at 30 days after hospitalization was significantly higher with low SCC at 24 h than with high SCC at 24 h. Notably, these results were obtained at a primary care animal hospital and involved early disease stages before treatment initiation.

Schoeman et al. reported that, in dogs with parvoviral diarrhea, the SCC values on Days 2 and 3 were significantly higher in the non-survivor group than in the survivor group [17]. Although these results were based on a specific disease, they were similar to our results. Therefore, measuring the SCC at 24 h after the initiation of treatment appeared to be important. Within 24 h of hospitalization, it may be crucial to relieve stress, including the stress of hospitalization.

The neutrophil count, lymphocyte count, and blood glucose concentration had been considered to be stress markers in dogs [27, 28], but these were not significant prognostic markers in this study. Due to the fact that neutrophils and lymphocytes are rapidly transported to tissues, the corresponding cell counts may not be accurately reflected in the circulating blood; neutrophil and lymphocyte counts may be useful stress markers in chronic inflammation but not in acute inflammation [29]. In the present study, the CRP concentration was measured, because SCC and inflammatory cytokines (e.g., interleukin-1 [IL-1], IL-6, and tumor necrosis factor-alpha) are known to increase after stimulation, such as trauma [30]. The results showed that the concentration of CRP was increased in several cases and that many of the medical diseases were in the acute phase. Therefore, measuring the neutrophil and lymphocyte counts in the circulating blood may not be useful in acute-phase diseases. Further, blood glucose concentration was not shown to be useful in this study, probably because our population included cases of diabetes and infectious diseases. In humans and dogs, CRP is known to be a useful marker for predicting the prognosis of specific inflammatory diseases [31–34]; however, this study showed otherwise, and this result may be attributed to the inclusion of cases other than inflammatory diseases. We stratified the dogs into high-CRP and low-CRP groups and examined whether the SCC predicted the prognosis without the effect of CRP. As a result, SCC showed no significant difference in the high-CRP group; however, there was a significant difference in the low-CRP group (data not shown). Hence, SCC could be an independent prognostic factor. Therefore, compared with CRP, SCC may be a more sensitive prognostic indicator.

During severe illnesses, several factors can impair the normal corticosteroid response. These factors include preexisting conditions that affect the HPA axis; however, corticosteroid insufficiency during the course of acute illness can also be due to abnormal responses to corticotropin-releasing hormone and corticotropin induced by head injuries, central nervous system depressants, or pituitary infarction; adrenal hemorrhage caused by septicemia and underlying coagulopathy; extensive destruction of adrenal tissues caused by tumors or infections; and direct inhibition of adrenal cortisol synthesis caused by high levels of inflammatory cytokines in patients with sepsis [35]. Adrenal insufficiency in dogs was reported to occur in acute illnesses such as sepsis, severe trauma, and gastric dilatation-volvulus [36]. In our study, we did not determine the presence of adrenal insufficiency because we did not perform an adrenocorticotropic hormone stimulation test. Due to the fact that adrenal insufficiency can be misdiagnosed on the basis of SCC measurement alone, a method to distinguish cases of adrenal insufficiency is necessary in future studies.

Our findings suggested that only SCC at 24 h after hospitalization had a significant independent influence on mortality. Because there was no suitable reference for calculating the sample size of this study, the effect size was calculated [37]. From the results obtained through this study, the effect size was *h* = 0.75. Based on the large effect size and high detection rate, the number of samples in this study was considered to be sufficient. Older age, which is associated with many complications, was also hypothesized to be a factor that could affect mortality; however, it was not correlated with mortality in this study. From these results, it was observed that SCC at 24 h is an independent prognostic factor not affected by age. In this study, euthanasia was performed in one case that developed hemothorax due to a tumor that was assessed to be challenging to treat. Therefore, it was not likely to be involved in the outcome on day 30.

Tarjanyi et al. reported that, compared with total cortisol measurements at 0 and 6 h after hospitalization, critical illnesses were more useful in predicting the risk of mortality in human patients [18]. In the present study, cortisol was measured at 0 and 24 h after hospitalization. Because our subjects were dogs, which are small animals, it would have been unethical to conduct frequent blood tests. Therefore, 0 and 24 h were selected as time points, considering the influence of individual diurnal variations in Inc-SCC and R-Inc-SCC. No evaluation at 6 h was performed, although this might have been more useful. In the present study, the follow-up period was 30 days after the hospitalization, and this was based on a human study [25], because there was no available veterinary medicine reference. Considering the average healing period of various internal medical conditions in dogs, the number of days of follow-up in this study was considered to be appropriate.

In humans with severe infections, traumas, burns, illnesses, or surgeries, the production of cortisol can increase by as much as six times and is partially proportional to the severity of the illness [35, 38–40]. Diurnal variations in cortisol secretion are known in humans [41]. However, during severe illnesses, these diurnal variations in cortisol secretion are not observed [42]. On the other hand, dogs do not usually have fixed diurnal variations, wherein cortisol secretion rises during sleep; however, individual diurnal variations have been observed [43, 44].

The primary limitation of this study was that it was a small-scale study with a relatively small sample size. Moreover, majority of the dogs raised in the area where our hospital was located were of small breeds; our results may be applicable to small dogs alone. Our results may also have relevance to primary care animal hospitals that handle various medical diseases, but it would be necessary to extensively investigate specific conditions in a larger number of samples to enable clinical application. Finally, knowing that a high SCC may affect subsequent treatments, veterinarians might need to be blinded to the results of SCC in future studies.

## Conclusions

SCC as a stress marker at 24 h after hospitalization is a useful prognostic marker in dogs requiring hospitalization at a primary care animal hospital. However, further studies are needed to evaluate the precision and utility of the calculated 6.6 μg/dL SCC cutoff in this study. Our results need to be validated on a large number of samples using specific disease conditions.

## Data Availability

The datasets analyzed during the current study are available from the corresponding author upon reasonable request.

## Abbreviations

CBC: Complete blood count
CI: Confidence interval
CRP: C-reactive protein
HPA: Hypothalamic–pituitary–adrenal
HR: Hazard ratio
IL: Interleukin
Inc-SCC: Increase in SCC from 0 to 24 h
IQR: Interquartile range
R-Inc-SCC: Rate of increase in SCC from 0 to 24 h
ROC: Receiver operating characteristic
SCC: Serum cortisol concentration

## Footnotes

1 Celltac alpha; Nihon Kohden, Tokyo, Japan

2 FUJI DRI-CHEM 7000 V; FUJIFILM Corporation, Tokyo, Japan

3 IMMUNO AU1 0 V; FUJIFILM Corporation, Tokyo, Japan

4 IMMULITE 1000; Siemens Healthcare Diagnostics, Eschborn, Germany
